# The effectiveness of an online short-format Recovery College model: a co-learning model to support mental health

**DOI:** 10.1186/s13033-024-00637-7

**Published:** 2024-05-03

**Authors:** Catherine Briand, Charles-Édouard Giguère, Julio Macario de Medeiros, Catherine Vallée, Francesca Luconi, Brigitte Vachon, Marie-Josée Drolet, Johana Monthuy-Blanc, Amani Mahroug, Régis Hakin

**Affiliations:** 1 Department of Occupational Therapy, University of Quebec at Trois-Rivières, Trois-Rivières, Québec Canada; 2 Research Center of Institut universitaire en santé mentale de Montréal, Montréal, Québec Canada; 3 School of Rehabilitation Sciences, Université Laval, Québec City, Québec Canada; 4 Office for Continuing Professional Development, Faculty of Medicine and Health Sciences, McGill University, Montréal, Québec Canada; 5 School of Rehabilitation, Faculty of Medicine, Université de Montréal, Montréal, Québec Canada; 6 Department of Education, University of Quebec at Trois-Rivières, Trois-Rivières, Québec Canada

**Keywords:** Co-learning, Co-production, Covid-19 pandemic, Effectiveness, Linear mixed models, Mental health, Online training, Recovery College

## Abstract

**Background:**

Our societies are facing mental health challenges, which have been compounded by the Covid-19. This event led people to isolate themselves and to stop seeking the help they needed. In response to this situation, the Health and Recovery Learning Center, applying the Recovery College (RC) model, modified its training program to a shorter online format. This study examines the effectiveness of a single RC training course delivered in a shortened online format to a diverse population at risk of mental health deterioration in the context of Covid-19.

**Methods:**

This quasi-experimental study used a one-group pretest-posttest design with repeated measures. Three hundred and fifteen (*n* = 315) learners agreed to take part in the study and completed questionnaires on wellbeing, anxiety, resilience, self-management, empowerment and stigmatizing attitudes and behaviors.

**Results:**

Analyses of variance using a linear mixed models revealed that attending a RC training course had, over time, a statistically significant effect on wellbeing (*p* = 0.004), anxiety (*p* < 0.001), self-esteem/self-efficacy (*p* = 0.005), disclosure/help-seeking (*p* < 0.001) and a slight effect on resilience (*p* = 0.019) and optimism/control over the future (*p* = 0.01).

**Conclusions:**

This study is the first to measure participation in a single online short-format RC training course, with a diversity of learners and a large sample. These results support the hypothesis that an online short-format training course can reduce psychological distress and increase self-efficacy and help-seeking.

**Trial registration:**

This study was previously approved by two certified ethics committees: *Comité d’éthique de la recherche du CIUSSS EMTL*, which acted as the committee responsible for the multicenter study, reference number MP-12-2021-2421, and *Comité d’éthique avec les êtres humains de l’UQTR*, reference number CER-20-270-07.01.

## Background

Our societies are facing major mental health challenges [1–3]. These challenges have been compounded by the Covid-19 pandemic [4–7]. While all citizens were affected, vulnerable populations such as women, students, gender-diverse individuals, healthcare workers and people living with mental or chronic illnesses faced greater risks of mental health deterioration during Covid-19 [8–13]. High levels of anxiety and psychological distress have been documented for many at-risk groups and in the general population during Covid-19, and a higher demand for mental health services has been observed, an increase which has overwhelmed health systems [4, 5, 14, 15]. This situation has led people to isolate themselves and to refrain from seeking help [16, 17], situation not unique to the Covid-19, and can also occur in other contexts, for example, with people with restricted mobility, in isolated areas, etc. It was in the Covid-19 context that the Health and Recovery Learning Center, applying the Recovery College (RC) model, decided to modify its training program, adapting its in-person courses to a shortened online format, in order to quickly reach as many people as possible [18, 19].

Launched in England in 2009, Recovery Colleges (RC) are training centers that offer free training courses (in a co-learning workshop format) on mental health, well-being, recovery and living well together [20, 21]. Now offered worldwide (in 28 countries), they propose a new way of intervening on mental health based on principles of mutual learning and recognition of experiential knowledge [22, 23]. In recent years, the RC model has been the subject of several studies. Fifty-nine studies, published between 2013 and 2023, have been identified confirming the positive outcomes of the model [24]. In particular, the RC model improves mental health knowledge, self-regulation skills, empowerment, self-worth, mental health wellbeing, personal growth and recovery, social connectedness, the use of recovery-oriented practices (e.g. power dynamics between health professionals and service users, strengths-based approach, families’ involvement, etc.), beliefs and prejudices towards mental health, and reduces health care utilization [20, 25–35]. However, these studies were carried out on in-person courses, of long duration and with the possibility of participating in more than one course (and not for a single online short-format training course). In addition, published quasi-experimental outcome studies are based on small samples of 19 to 58 participants [26, 29, 34, 36]. Only two outcome studies exceeded these numbers of participants: the study by Sutton & French [37] exclusively with healthcare professional learners (*n* = 135) and the study by Durbin et al. [38] exclusively with homeless learners (*n* = 92). Also, outside outcome studies, three studies on the use of health services involving exclusively people living with mental illness have larger sample sizes of over 100 participants [25, 31, 39]. In other words, no experimental studies have yet been published on the outcomes of online short-format training courses with a large and diverse sample of learners (apart from exploratory analyses at the Health and Recovery Learning Center [18, 19]).

The aim of this study, using a pre-post quasi-experimental design, is to evaluate the outcomes of participation in a single online short-format course. The variables measured are: (i) wellbeing; (ii) anxiety; (iii) resilience; (iv) self-management strategies; (v) empowerment; and (vi) stigmatizing attitudes and behaviors, for all the various learners taking part in an RC training course. Given the context of Covid-19, measures of anxiety and resilience were added to the usual measures used in RC studies [4, 24].

## Methods

### Study design

This quasi-experimental study used a one-group pretest-posttest design with repeated measures. Baseline data collection (T0) took place during the registration period and before the training sessions (one day to three weeks before). Outcomes were assessed immediately after the end of the training sessions and up to 3 weeks later (T1), and there was a final follow-up after 3 months (between 12 and 14 weeks) (T2). These 3-week completion periods at each measurement time were determined to allow as many learners as possible to participate in the study within a reasonable timeframe without pressure.

The research question was: Does participation in an online short-format RC course lead to improvements in (i) wellbeing, (ii) anxiety, (iii) resilience, (iv) clinical, empowerment and vitality self-management strategies, (v) empowerment (self-esteem/self-efficacy, power-powerlessness, community activism and autonomy, optimism and control over the future, righteous anger), and (vi) stigmatizing attitudes and behaviors (attitudes towards people living with mental illness, disclosure/help-seeking, social distance) among the various learners? The hypothesis is that the shortened online adaptation of the RC model leads to benefits for learners across all the variables studied.

### Intervention

Since fall 2019, the Health and Recovery Learning Center (*Centre d’Apprentissage Santé et Rétablissement* - CASR), the only RC in the province of Quebec, has been offering training courses (in a co-learning workshop format) to the entire population of the province. In the fall of 2020, in response to the Covid-19 pandemic, CASR adapted all its training to online short-format courses in order to quickly reach as many people as possible. Those most at risk of mental health deterioration in the context of Covid-19 (women, college and university students, healthcare workers, people living with mental or chronic illness, family caregivers) as well as members of the general population were the target groups [8–13]. Quebec is Canada’s only solely French-speaking province, with a population of over 8 million and a large surface area of 1.7 million square kilometers.

The courses were publicized by partner organizations representative of these target groups (healthcare institutions, colleges and universities, community mental health organizations, patients’ and family caregivers’ associations, etc.) as well as via CASR’s website and social networks. From fall 2020 to spring 2022, CASR offered 80 online courses lasting 6 h (three 2-hour periods) to 1173 different learners (using the Zoom® platform). For each online course, the target audience was constituted as to include a diversity of learners (12–18 diverse learners per course).

RC training courses are distinguished by: (i) the diversity backgrounds of the learners and trainers (people with lived experience of mental health disease, relative of a person with a mental health disease, peer helpers, educational and health professionals, administrative staff, manager and director in educational and health systems, citizens, etc.); (ii) the hybridization and cross-fertilization of knowledge (theoretical, clinical, practical, and experiential) through participatory methods and active pedagogies; (iii) the promotion of egalitarian social relationships free of judgment, where speaking out is encouraged [40–42]. The RC model is based on a genuine commitment to co-production and co-learning where lived experience and professional experience are placed on an equal level, offering learners from diverse backgrounds the opportunity to learn from each other [20, 21]. The foundations of the model are based on the fundamental equality of knowledge and human beings, the equitable participation of learners (including the RC team i.e., trainers and partner organizations) and the experience of egalitarian relationships free of prejudice [40, 43].

### Recruitment and ethical consent

Recruitment was carried out according to a convenience sampling procedure, drawing from all learners participating in CASR training courses delivered from fall 2020 to spring 2022 (see Table 1 for details of training sessions). Potential participants received information about the study by email from the research coordinator. In line with the RC model, minimal eligibility criteria were set, namely: being at least 16 years old and being able to attend an online meeting (in terms of technical equipment, computer skills and sensory and cognitive abilities). No exclusion criteria were applied. Participation was entirely voluntary, and no financial compensation was offered (apart from the possibility of winning a gift certificate on completion of the course). From a total number of 1173 learners registered in the training courses that were invited to participate in the study, 315 (27%) signed an informed consent form attesting to their agreement to the data collected being used for research. The study and the informed consent form had been previously approved by a certified ethics committee (*Comité d’éthique de la recherche du CIUSSS EMTL*, which acted as the committee responsible for the multicenter study, reference number MP-12-2021-2421). The ethics committee of the principal investigator’s (CB) university has also given its approval (*Comité d’éthique avec des êtres humains de l’UQTR*, reference number CER-20-270-07.01).

**Table 1:** Distribution of participants by training sessions

Training Sessions	T0 (*n* = 261)	T1 (*n* = 227)	T2 (*n* = 205)
Fall 2020	51	45	47
Spring 2021	64	57	44
Fall 2021	46	42	38
Winter 2022	57	52	47
Spring 2022	43	31	29

### Data collection procedures

An online survey link (with Google Forms platform) was e-mailed to participants at T0, T1 and T2 (before the training course, after it, and 3 months later for a follow-up), and targeted reminders were sent to those who had not yet completed the survey. Relevant sociodemographic information was also collected at T0 such as gender, age, country of birth, language spoken at home, level of education, main occupation/type of learner, type of mental health knowledge (i.e., experiential, clinical, theoretical), lifetime diagnosis of mental illness, mental health services received in the last 6 months.

Of the 315 learners who signed the research consent form, 261 (83%) participants completed the baseline survey at T0 (17% attrition). At T1, 227 participants completed the survey (additional 13% attrition). At the T2 follow-up, 205 participants completed the survey (additional 10% attrition). Of these, 38 learners were excluded, as they had responded to the survey after the deadline or joined the RC team as trainers. The distribution of participants by training sessions is presented in Table 1.

### Outcome measures

Participants completed a set of questionnaires to assess the intervention outcomes at T0, T1 and T2: the *Warwick-Edinburgh Mental Wellbeing Scale - short form (WEMWBS)* [44], the *Generalized Anxiety Disorder-7 (GAD-7)* [45], the *Connor-Davidson Resilience Scale (CD-RISC-10)* [46], the *Mental Health Self-Management Questionnaire (MHSQ)* [47], the *Consumer Constructed Scale to Measure Empowerment* [48, 49], the *Opening Minds Scale (OMS)* [50, 51].

#### Wellbeing

Wellbeing was measured using the *Warwick-Edinburgh Mental Wellbeing Scale - short form (WEMWBS)* [44]. The WEMWBS is a 7-item measure that uses a five-point Likert scale to identify mental wellbeing and overall satisfaction with life. The WEBWBS shows excellent internal consistency (α from 0.89 to 0.91), good test-retest reliability (0.83) and includes a single factor.

#### Anxiety

Anxiety was measured using the *Generalized Anxiety Disorder-7 (GAD-7)* [45]. The GAD-7 is a 7-item measure that uses a four-point scale to identify the level of anxiety, including clinical and severe levels of anxiety. The GAD-7 shows excellent internal consistency (α = 0.92), good test-retest reliability (0.83) and includes a single factor. Using a cut-off score of 10, the GAD-7 has a sensitivity of 89% and a specificity of 82%.

#### Resilience

Resilience capacity was measured using the *Connor-Davidson Resilience Scale* (CD-RISC-10) [46]. The CD-RISC-10 is a 10-item measure that uses a five-point Likert scale to identify stress coping/adaptability skills. The CD-RISC-10 includes a single factor and shows good internal consistency (α = 0.85) and good test-retest reliability (0.81).

#### Self-management

Self-management strategies were measured using the *Mental Health Self-Management Questionnaire (MHSQ)* [47]. The MSHQ is an 18-item measure that uses a five-point Likert scale to identify self-management strategies used. The MSHQ includes three different factors (subscales): Clinical (getting help and using resources), Empowerment (building upon strengths and positive self-concept to gain control), Vitality (active and healthy lifestyle). The MSHQ shows good internal consistency (clinic α = 0.69, empowerment α = 0.81, vitality α = 0.75) and good test-retest reliability (clinic α = 0.78, empowerment α = 0.76, vitality α = 0.85).

#### Empowerment

Empowerment was measured using the *Consumer Constructed Scale to Measure Empowerment (CCSME)* [48, 49]. The CCSME is a 25-item measure that uses a four-point scale to identify the ability to regain power over one’s life. The CCSME includes five different factors (subscales): Self-esteem/self-efficacy, Power-powerlessness, Community activism and autonomy, Optimism and control over the future and Righteous anger. The CCSME shows poor to good internal consistency depending on the subscale (Self-esteem/self-efficacy α = 0.82, Power-powerlessness α = 0.59, Community activism and autonomy α = 0.59, Optimism and control over the future α = 0.45, Righteous anger α = 0.64).

#### Stigmatizing attitudes and behaviors

Stigma was measured using the *Opening Minds Scale for Health Care Providers (OMS-HCP)* [50, 51]. Although the questionnaire was designed for the HCP population, it was used for all the learners in our study. The OMS is a 15-item measure that uses a five-point Likert scale to identify stigmatizing attitudes and behaviors. The OMS includes three different factors (subscales): Attitude towards people with mental health, Disclosure/Help-seeking, Social Distance. The OMS shows good internal consistency (overall α = 0.79) (Attitude α = 0.68, Disclosure/Help-seeking α = 0.67, Social Distance α = 0.68).

### Data Analysis

All analyses were performed in R, version 4.2.1 [52]. Descriptive statistics were used to describe the sample, and linear mixed-effect models (LMM) were applied to assess change in outcomes over time, which refer to a “Effect of Time” (package lme4, [53]). The MLM model included “Time of data collection” as a categorical variable (i.e., T0, T1 and T2) and the session as a covariate (Fall 2020, Spring 2021, Fall 2021, Winter 2022, & Spring 2022) in the fixed effects and a random intercept. The overall “Effect of Time” was tested and when this effect of time was found statistically significant, change between pre (T1) and post (T2) intervention were tested using *post hoc* contrasts (T1-T0, T2-T0, T2-T1), adjusted for multiple comparisons using Tukey family-wise adjustments. To obtain a statistical power of 80% and a type 1 error of 0.05 (α = 0.05) for an effect size of f = 0.23 (Partial eta-sq.=0.05), a sample of 95 participants was required.

## Results

### Sample characteristics

Two hundred and sixty-one CASR learners (84% female gender, average age of 43.6 years, ranging from 21.3 years to 79.2 years) were recruited. Sociodemographic information is presented in Table 2.

**Table 2:** Sociodemographic information of participants at baseline

	Total Sample (*n* = 261)
**Gender**	
Female	218 (84%)
Male	40 (15%)
Non-binary	3 (1%)
**Age**	
Mean (SD)	43.7 (13.68)
Range	21.3–79.2
**Country of Birth**	
Canada	225 (86%)
France	16 (6%)
Other American countries (Haïti, Brazil, Chile, Colombia, Cuba)	7 (3%)
Other European countries (Belgium, Poland, Switzerland, Ukraine, Russia)	6 (2%)
Asian countries (China, Liban)	4 (2%)
African countries (Burkina Faso, Ivory Coast, Mauritania)	3 (1%)
**Home Language**	
French	245 (93%)
English	8 (3%)
Spanish	4 (2%)
Others (Creole, Portuguese, Russian, Pulaar)	4 (2%)
**Highest Level of Education** ^**1**^	
High school	13 (5%)
Professional courses	14 (5%)
College	48 (18%)
University certificate	12 (5%)
Bachelor’s degree	93 (36%)
Master’s degree	70 (27%)
Doctoral diploma	11 (4%)
**Type of Learner (Self-Assessed)**	
Healthcare workers from public system	68 (26%)
Administrative staff, manager, director of education or health	45 (17%)
College or university student	28 (11%)
Healthcare workers from non-profit organization	25 (10%)
Person with lived experience of mental health disease	24 (9%)
Citizen	21 (8%)
Educational professionals	14 (5%)
Relative of person with mental health disease	11 (4%)
Unknown answer	25 (10%)
**Mental Health Knowledge** ^**2**^	
Theoretical knowledge	192 (74%)
Experiential knowledge	187 (72%)
Clinical knowledge	143 (55%)
**Mental Health Parameters** ^**3**^	
Received a diagnosis of mental illness lifetime	121 (46%)
Received mental health services in last 6 months	103 (40%)

1: Education levels were from lowest to highest (and not in descending order of responses as for the other variables)

2: More than one type of mental health knowledge is possible per participant

3: Each mental health parameter is covered by a different question in the questionnaire. Answers are therefore mutually exclusive

### Longitudinal effect of time

Results concerning the effect of time are reported in Tables 3 and 4.

**Table 3:** Longitudinal effect of time

Outcome	F^1^	*p* ^2^	*p* adjusted^3^
**Wellbeing**	5.71	0.004**	-
**Anxiety**	7.68	< 0.001***	-
**Resilience**	4.01	0.019*	-
**Self-Management**			
Clinical	0.94	0.39	0.59
Empowerment	0.47	0.62	0.62
Vitality	3.29	0.038*	0.11
**Empowerment**			
Self-esteem/self-efficacy	6.84	0.001**	0.005**
Power-powerlessness	0.88	0.42	0.42
Community activism and autonomy	3.40	0.034*	0.06
Optimism and control over the future	5.61	0.004**	0.01*
Righteous anger	1.52	0.22	0.28
**Stigma**			
Attitudes towards people living with mental illness	0.17	0.85	0.99
Disclosure/help-seeking	16.39	< 0.001***	< 0.001***
Social Distance	0.01	0.99	0.99

^1^ : Analysis of variance, effect of time

^2^ : *p* value : * < 0.05; ** < 0.01; *** < 0.001

^3^ : *p* value adjusted for test multiplicity

**Table 4:** Change in mean score over time

Outcome	Baseline (T0) Mean (SE)	Post-Interv (T1) Mean (SE)	Follow-Up (T2) Mean (SE)	T1-T0 t value (*p*)^1^	T2-T0 t value (*p*)^1^	T2-T1 t value (*p*)^1^
**Wellbeing**	26.08 (0.21)	26.47 (0.21)	26.76 (0.22)	1.99 (*p* = 0.12)	3.34 (*p* = 0.003)	1.42 (*p* = 0.33)
**Anxiety**	5.73 (0.25)	5.18 (0.26)	4.82 (0.26)	-2.44 (*p* = 0.040)	-3.85 (*p* < 0.001)	-1.49 (*p* = 0.30)
**Resilience**	27.49 (0.35)	28.01 (0.36)	28.26 (0.37)	1.94 (*p* = 0.13)	2.73 (*p* = 0.018)	0.86 (*p* = 0.66)
**Self-Management**						
Clinical	8.33 (0.33)	8.08 (0.34)	8.01 (0.35)	-1.03 (*p* = 0.56)	-1.28 (*p* = 0.41)	-0.29 (*p* = 0.95)
Identity	23.91 (0.32)	24.00 (0.33)	24.19 (0.34)	0.33 (*p* = 0.94)	0.96 (*p* = 0.60)	0.64 (*p* = 0.80)
Vitality	10.50 (0.18)	10.25 (0.19)	10.65 (0.19)	-1.69 (*p* = 0.21)	0.94 (*p* = 0.62)	2.51 (*p* = 0.033)
**Empowerment**						
Self-esteem/self-efficacy	28.92 (0.22)	29.30 (0.23)	29.54 (0.23)	2.35 (*p* = 0.05)	3.62 (*p* < 0.001)	1.36 (*p* = 0.36)
Power-powerlessness	16.71 (0.12)	16.85 (0.12)	16.73 (0.13)	1.25 (*p* = 0.42)	0.18 (*p* = 0.98)	-1.00 (*p* = 0.58)
Community activism and autonomy	17.51 (0.11)	17.29 (0.11)	17.56 (0.11)	-2.06 (*p* = 0.10)	0.48 (*p* = 0.88)	2.40 (*p* = 0.044)
Optimism and control over the future	8.98 (0.08)	9.14 (0.09)	9.25 (0.09)	2.04 (*p* = 0.10)	3.30 (*p* = 0.003)	1.32 (*p* = 0.38)
Righteous anger	4.63 (0.08)	4.74 (0.08)	4.72 (0.09)	1.64 (*p* = 0.23)	1.28 (*p* = 0.41)	-0.28 (*p* = 0.96)
**Stigma**						
Attitudes towards people living with mental illness	11.04 (0.20)	11.07 (0.20)	11.14 (0.21)	0.19 (*p* = 0.98)	0.57 (*p* = 0.83)	0.38 (*p* = 0.92)
Disclosure/help-seeking	9.19 (0.17)	8.49 (0.17)	8.60 (0.18)	-5.34 (*p* < 0.001)	-4.29 (*p* < 0.001)	0.78 (*p* = 0.72)
Social Distance	8.13 (0.14)	8.15 (0.16)	8.13 (0.17)	0.14 (*p* = 0.99)	-0.01 (*p* = 1.00)	-0.14 (*p* = 0.99)

^1^ : *p* value : < 0.05: *; < 0.01:**; < 0.001: ***

#### Wellbeing

A statistically significant overall effect of time was found (F(2,434) = 5.71, *p* = 0.004). A statistically significant contrast between T0 and T2 (t = 3.34, *p* = 0.003) indicates a progressive and continuous increase in wellbeing scores, with effects observed only after three months.

#### Anxiety

A statistically significant overall effect of time was found (F(2,437) = 7.68, *p* < 0.001). A statistically significant contrast between T0 and T2 (t = 3.85, *p* < 0.001), and a slight contrast between T0 and T1 (t = 2.44, *p* = 0.040) indicate a steady reduction in participants’ anxiety level over time (during and after the course).

#### Resilience

A slight overall effect of time was found (F(2,429) = 4.01, *p* = 0.019). A slight contrast between T0 and T2 (t = 2.73, *p* = 0.018) indicates a small progressive and continuous increase in resilience scores.

#### Self-management

After adjusting the statistical significance level for test multiplicity, no overall effect of time was observed for any of the subscales of self-management (Clinical Self-Management, Identity and Vitality).

#### Empowerment

After adjusting the statistical significance level for test multiplicity, a statistically significant overall effect of time was found for the Self-esteem/self-efficacy subscale (F(2,430) = 6.84, *p* = 0.001, *p* adjusted = 0.005). A statistically significant contrast between T0 and T2 (t = 3.62, *p* < 0.001), and a slight contrast between T0 and T1 (t = 2.35, *p* = 0.05) indicate a steady reduction during and after the course. Also, a slight overall effect of time was observed for the Optimism and control over the future subscale (F(2,439) = 5.61, *p* = 0.004, *p* adjusted = 0.01). A statistically significant contrast between T0 and T2 (t = 3.30, *p* = 0.003) indicates a progressive and continuous increase in Optimism and control over the future subscale, with effects observed only after three months. No statistically significant effect was found for any other subscales (Community activism and autonomy, Power-powerlessness and Righteous anger).

#### Stigmatizing attitudes and behaviors

After adjusting the statistical significance level for test multiplicity, a statistically significant overall effect of time was found for the Disclosure/help-seeking subscale (F(2, 434) = 16.39, *p* < 0.001, *p* adjusted < 0.001). A statistically significant contrasts between T0 and T1 (t = 5.34, *p* < 0.001) and between T0 and T2 (t = 4.29, *p* < 0.001) indicates a steady reduction of stigmatizing attitudes and behaviors during and after the course. No overall effect of time was observed for the Attitudes towards people living with mental illness and Social Distance subscales.

## Discussion

The objective of this study was to examine the effectiveness over time of an online short-format RC training course. As expected, the results revealed an effect of time on wellbeing, anxiety, self-esteem/self-efficacy, disclosure/help-seeking and a slight effect of time on resilience and optimism/control over the future. A steady change during and after the course was observed in anxiety, self-esteem/self-efficacy and disclosure/help-seeking, and a progressive and continuous increase, with effects manifesting only after three months, was observed in wellbeing, resilience, and optimism/control over the future.

These results are aligned with previous studies on the RC model, which looked at in-person courses, and confirm the relevance of online short-format training courses delivered to a diversity of learners. These results concur with the study by Wilson et al. [34], which confirmed the increase in confidence, and the reduction in anxiety and social isolation in learners. These results are also aligned with the studies by Ebrahim et al. [26] and Meddings et al. [28], which demonstrated improvements in learners’ well-being, personal resources, and psychological distress. Sommer et al. [30] suggested that RC training courses leads to increased hopefulness for the future as well as changes in attitudes, which was also confirmed by Perkins & Repper [20]. As indicated by Nurser et al. [29], learners feel less self-stigma following participation in RC training courses.

These results are also aligned with the findings of the published qualitative study carried out at the CASR [18]. Three themes emerging from the thematic analysis are consistent with the observed outcomes. After a RC course, learners mentioned: (1) taking better care of themselves and their mental health (managing stress better and feeling more resilient and confident), (2) changing how they look at themselves (becoming self-aware), and (3) improving and modifying their behaviors (in particular, reflecting on how we deal with our prejudices and stigmas).

This quasi-experimental study is the first to measure the effect of participation in a single online short-format training course, with a diversity of learners and a large sample. These initial results support the possibility that a short RC-type online training course can slow the progression of psychological distress and prevent the deterioration of mental health and the aggravation of mental disorders in the general population and at-risk groups. After six hours of training, learners experienced a reduction in their anxiety levels. They increased their self-esteem/self-efficacy and their ability to seek help (increased self-disclosure). After three months post-training, they showed improved well-being, resilience and optimism/control over the future. These results open the way to further possibilities for the Recovery College model. That said, as mentioned by Ali et al. [54], it is important to have a clear understanding of the model, to reproduce the original model’s mechanisms of action and thus, achieve the expected outcomes. Establishing an egalitarian learning space is fundamental to the model’s effectiveness [20, 21, 40, 42]. The CASR team has rigorously followed the principles of the RC model, despite the shortened online adaptation. The trainers received over 30 h of basic training, over 15 h of additional training for the online adaptation, and they regularly participate in a trainers’ community of practice to ensure that the principles of the model are respected. For example, techno-pedagogies have been put in place to ensure active mutual learning between learners, opportunities for exchange and self-assertiveness (speaking up) in which everyone’s voice is respected equally. Also, the intentional mixing of learners and trainers in each group ensured the presence of different types of knowledge (theoretical, clinical, practical, experiential). Each group enabled direct contact between professionals and people/relatives living with mental illness in a safe place of mutual recognition free from judgment. In this way, it is possible to co-construct together a unified framework of understanding and create integrated knowledge that pools the diverse knowledge of all learners. By respecting the principles and mechanisms of action of the RC model, we can create a truly transformative learning environment.

Finally, these findings pave the way for short, effective mental health education interventions that empower people and make a difference to psychological distress. As part of the global effort to improve mental health intervention, these short interventions should be added to the mental health care “toolkit”, as well as to initiatives for more rapid referral to appropriate resources [55–57].

### Limits and future perspective

This study has certain limitations. Firstly, it would have been interesting to have a design with a control group representative of the study population. Also, as the focus was to measure outcomes in a diversity of learners, it was not possible to distinguish effects by learner profile (professionals vs. people with lived experience of mental health disease vs. university students) or by age and gender. The next steps should enable analysis by learner type or cluster analysis. For this, a sample of over 300 participants is required. Also, despite their diversity, the learners represented the usual or traditional RC population, considering that this was an online adaptation. Greater diversity could be achieved, with better representation of men, ethnic minorities, and gender-diverse populations. Furthermore, as the measure of anxiety is very sensitive to change, we may wonder whether the effects observed are caused by the intervention or by changes in the Covid-19 situation. The next step is probably to reproduce these results in a context outside Covid-19, and compare face-to-face vs. online courses. We could also add other measurement times, for example during and after the intervention, to better understand the changes observed. Despite these limitations, this study is the first experimental study to measure participation in a single online short-format RC training courses with a large and diverse sample of learners.

## Conclusion

This quasi-experimental study is the first to investigate the effects of an online adaptation of the RC model, with a diversity of learners and a large sample. The statistically significant effects of time on wellbeing, anxiety, self-esteem/self-efficacy, disclosure/help-seeking, as well as on resilience and optimism/control over the future are very promising for the evolution of the RC model and for co-learning models integrating all types of knowledge. These results support the possibility that online short-format RC training courses can slow the progression of psychological distress and prevent the deterioration of mental health through an increased sense of self-efficacy and help-seeking. These findings pave the way for effective short mental health education interventions that empower people and make a difference to the level of psychological distress.

## Data Availability

The datasets generated during the present study are available from the corresponding author C.B. upon reasonable request. The datasets are not publicly available owing to privacy or ethical restrictions.
